# Association of dietary habits with general and abdominal obesity in Korean children and adolescents: cluster analysis of nationwide population survey data

**DOI:** 10.3389/fendo.2024.1424761

**Published:** 2024-09-04

**Authors:** Ye-Jin Yun, Yu-Jin Kwon, Yaeji Lee, Seok-Jae Heo, Ji-Won Lee

**Affiliations:** ^1^ Department of Medicine, Yonsei University College of Medicine, Seoul, Republic of Korea; ^2^ Department of Family Medicine, Yongin Severance Hospital, Yonsei University College of Medicine, Gyeonggi, Republic of Korea; ^3^ Division of Biostatistics, Department of Biomedical Systems Informatics, Yonsei University College of Medicine, Seoul, Republic of Korea; ^4^ Department of Family Medicine, Severance Hospital, Yonsei University College of Medicine, Seoul, Republic of Korea; ^5^ Institute for Innovation in Digital Healthcare, Yonsei University, Seoul, Republic of Korea

**Keywords:** child obesity, diet, lifestyle, nutritional education, clustering analysis

## Abstract

**Introduction:**

Childhood obesity is a growing global health concern, but few studies have investigated dietary factors specifically related to obesity and abdominal obesity in children and adolescents. Herein, we aimed to identify the dietary factors affecting childhood obesity in Korean children and adolescents.

**Methods:**

Data from the Korea National Health and Nutrition Survey (KNHANES) VIII were analyzed using K-means clustering analysis to identify distinct clusters based on nine variables related to dietary habit, nutritional status, and nutritional education. Multiple logistic regression analysis was used to examine the association between incident obesity risk and the different clusters. We enrolled 2,290 participants aged 6-18 years, and separated them into two distinct clusters; Healthy and Unhealthy Dietary Habit Groups, clusters 1 and 2, respectively.

**Results:**

Cluster 1 was characterized by a lower obesity prevalence, healthier dietary habits (regular breakfast consumption; fruit and vegetable, reduced total energy, and lower protein and fat intakes), and greater nutritional education than Cluster 2. After adjusting for confounders, compared with Cluster 1, Cluster 2 demonstrated a significantly higher prevalence (OR [95% CI]) of both general and abdominal obesity (1.49 [1.05–2.13], p=0.027 and 1.43 [1.09–1.88], p=0.009).

**Discussion:**

Maintaining optimal dietary quality and patterns are crucial to prevent childhood obesity. Further research is warranted to explore specific dietary interventions tailored to different clusters to effectively address childhood obesity.

## 1 Introduction

The World Obesity Atlas 2023 (1) shows a significant increase in the prevalence of obesity, particularly among children and adolescents worldwide; among boys, prevalence is estimated to double from 10% in 2020 to 20% in 2035 whereas, for girls, the prevalence is expected to similarly increase from 8% to 18% (1). This highlights the need for strategies to address the escalating burden of obesity, especially among younger populations, to mitigate long-term health consequences and the associated socioeconomic impacts.

Pediatric obesity not only poses a higher risk of sustained obesity, but also carries future health risks in adulthood that have been well-documented (2). The severity of obesity in children and adolescents is closely linked to a higher risk of metabolic syndrome (MetS) (3). Compared to normal-weight individuals, those who are overweight or obese have a 5 and 23 fold higher risk of MetS, respectively (4). Furthermore, childhood BMI has been associated with risks of diabetes, cancer, and cardiovascular diseases, even independent of adult BMI (5).

The treatment of obesity includes behavioral changes in diet, physical activity, sedentary behaviors, and sleep habits (6). The World Health Organization (WHO) recently suggested that limiting energy intake from total fats and sugars by increasing the consumption of fruits, vegetables, whole grains, and nuts, as well as engaging in regular physical activity, are highly recommended at the individual level for obesity prevention (7). It has further been well-documented that healthy dietary patterns are beneficial for children’s health (8, 9). Additionally, unlike in adults, children’s dietary habits are highly influenced by familial (parental) (10) and socioeconomic factors (11). As dietary habits are important for the prevention and treatment of childhood obesity, dietary factors that can predict obesity and MetS in Korean children and adolescents need to be identified.

Park et al. (12) previously investigated the association of dietary quality with body mass index (BMI) in obese children, but found no significant associations of dietary patterns and quality with BMI in obese children. However, the authors observed an association of high fat intake with weight gain in this population. Kim et al. (13) observed that children who participated in the school lunch program consumed more appropriate nutrients than those in the non-school lunch and skipping lunch groups. Moreover, they found that the school lunch group was less likely to become obese than the skipping lunch group.

Nonetheless, few studies have investigated dietary factors specifically related to obesity and abdominal obesity in children and adolescents. Therefore, in this study, using the K-means clustering algorithm, we aimed to identify dietary factors that increase the risk of obesity in children and adolescents in the Republic of Korea. Unlike in previous studies, we included various dietary habits, such as breakfast eating, frequency of fruit or vegetable consumption, eating out, proportion of macronutrients, nutritional education, and other demographic factors in order to create a comprehensive background for personalized prediction and management of childhood obesity in the Republic of Korea.

## 2 Materials and methods

**Figure 1:**
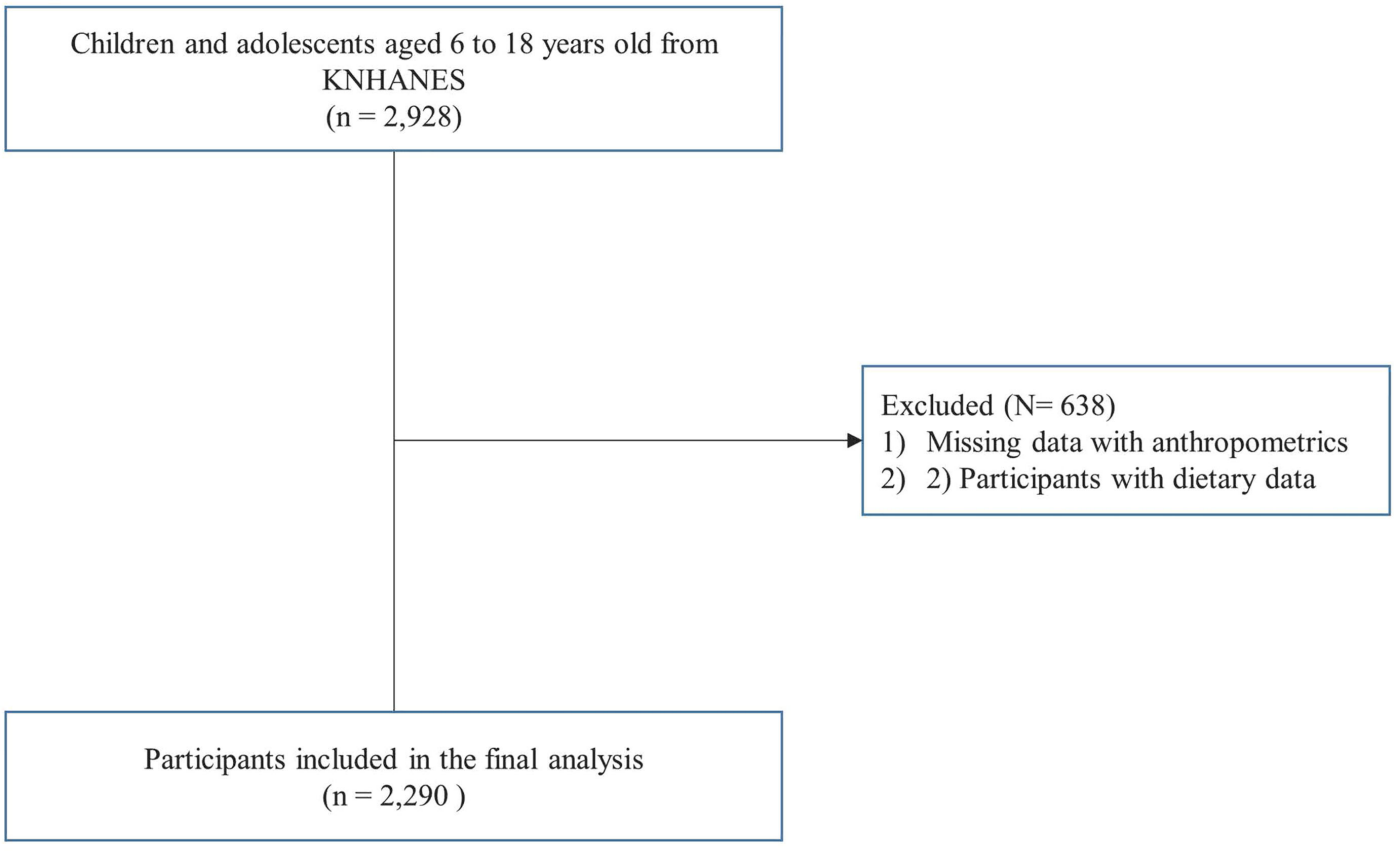
Flow chart of the study population.

The Korea National Health and Nutrition Examination Survey (KNHANES) is a cross-sectional survey that has been conducted annually by the Korea Centers for Disease Control and Prevention (KCDC) since 1998 to derive a comprehensive understanding of the health and nutritional status of the South Korean population. The KNHANES targets non-institutionalized Korean citizens residing in Korea, and follows a multistage, clustered probability design for sampling; detailed information on the KNHANES is available at: https://knhanes.cdc.go.kr/knhanes/eng. In this study, we used data from the KNHANES VIII (2019–2021). Of the 2,928 KNHANES VIII participants aged 6–18 years, those without anthropometric and dietary behavior data (n = 638) were excluded, and a total of 2,290 participants were included in the final analysis, as shown in the study flowchart in **Figure 1**. All participants provided written informed consent for the use of their data for research purposes. The study protocol was approved by the institutional review board of the Severance Hospital (approval no. 4-2022-0796).

### 2.1 Anthropometry and the adiposity index

In the KNHANES VIII, anthropometric measurements, including blood pressure (BP), height, weight, BMI, and waist circumference (WC), were measured by well-trained medical staff. BP was measured three times in the sitting position, and the average of the secondary and tertiary measurements was used in the analysis. Height was measured using a portable stadiometer, with accuracy to the nearest 0.1 cm, while weight was determined using a digital scale, accurate to the nearest 0.1 kg. During measurement, participants were advised to wear light attire and no shoes. WC was measured using a standard measuring tape at the narrowest point of the body, located between the lowest rib and the iliac crest. BMI was calculated by the dividing weight (in kilograms) by the square of height (in meters). The waist-to-height ratio (WHtR) was subsequently determined by dividing each individual’s waist circumference (WC) by height. For comparisons, the standard scores (z-scores) for BMI, WC, and WHtR were derived from the KNHANES VIII (2019-2021) using the method described by Kim et el (14), taking into account age and sex. Obesity was defined as BMI values above the 95th percentile, corresponding to age and sex categories following the Korea Centers for Disease Control and Prevention (KCDC) criteria (14). Abdominal obesity was defined as a WC that exceeded the 90th percentile according to age- and sex-specific criteria (15). Abdominal obesity based on WHtR was defined as WHtR ≥0.5 (16).

### 2.2 Dietary behaviors and nutritional education assessment

According to a standardized protocol, the dietary behaviors of children and adolescents were assessed by well-trained nutritionists through questionnaires that assessed the following items: breakfast frequency per week in the last year (almost every day, 1-4 times a week, or rarely); frequency of eating out (almost every day, more than once a week, or rarely); experience of nutritional education in the past year (yes/no); frequency of consuming vegetables (excluding kimchi and pickled vegetables), mushrooms, and seaweed in the past year (more than three times a day, once or twice a day, or less than once a day); and frequency of consuming fruits in the past year (>7, 2–6, or <1 time(s) per week). The total calorie intake and grams of carbohydrate, fat, and protein were calculated from the 24-hour dietary recall. The total consumption of carbohydrates, protein, and fat was subsequently converted to energy intake in calories (1 g carbohydrates = 4 kcal; 1 g protein = 4 kcal; and 1 g fat = 9 kcal). The proportion of carbohydrates, protein, and fat intake was calculated as follows: carbohydrate, protein, and fat intake calories/total calorie intake × 100.

### 2.3 Clustering analysis

K-means was used to form clusters using the nine variables related to dietary habit, nutritional status, and nutritional education (frequency of breakfast consumption, frequency of dining out, experience of nutritional education, frequency of consuming vegetables on average, frequency of consuming fruits on average, total calorie intake, proportion of carbohydrate intake, proportion of protein intake, and proportion of fat intake). The frequency of dietary behaviors was clustered by considering the categorical variables as continuous variables. K-means clustering was then performed on the standardized values to have zero mean and unit variance. Using the silhouette method 18, we determined the optimal number of clusters for dietary habits.

### 2.4 Statistical analysis

All data are reported as the mean ± standard deviation (SD) for continuous variables, or as frequency (proportion) for categorical variables. To compare the differences between clusters, we conducted independent t-tests for continuous variables and the Fisher’s exact test for categorical variables. Linear and logistic regression were applied to determine association between clusters of dietary habits and adiposity for Korean children and adolescents. In the regression analysis, age and sex were adjusted to reduce the confounding effects. Subgroup analysis was performed for age groups (6–12 and 13–18 years) and sex. All statistical analyses were conducted using R version 4.1.1 (R Foundation for Statistical Computing, Vienna, Austria). Statistical significance was set at a p-value of less than 0.05.

## 3 Results

### 3.1 Clinical characteristics of the two clusters

**Figure 2:**
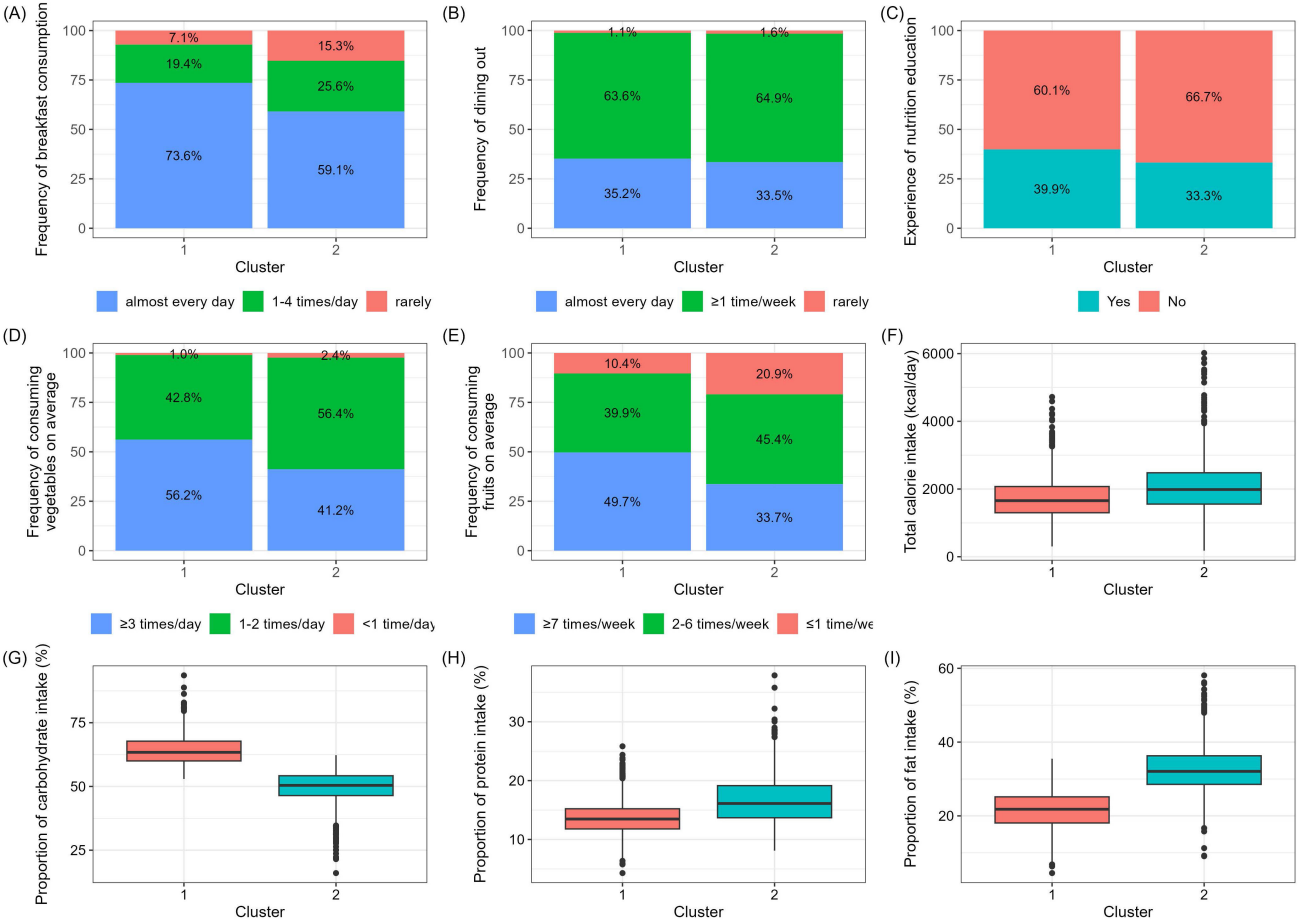
Characteristics of the two patient clusters. Data are presented as percentages or box plot (median, IQR). **(A)** Frequency of breakfast consumption, **(B)** Frequency of dining out, **(C)** Experience of nutrition education, **(D)** Average frequency of consuming vegetables, **(E)** Average frequency of consuming fruits, **(F)** Total calorie intake from 24-hour dietary recall, **(G)** Proportion of carbohydrate intake, **(H)** Proportion of protein intake, **(I)** Proportion of fat intake.

Using the K-means clustering algorithm, we produced two clusters from the overall participants (N = 2,290). Each cluster comprised participants with characteristics similar to the nine variables within the cluster. The distribution of the participants and characteristics of the two clusters are shown in **Figure 2**, **Table 1**. In total, 706 boys and 694 girls were grouped into Cluster 1 (N = 1,400), whereas 500 boys and 390 girls were grouped into Cluster 2 (N = 890).

**Table 1:** Clinicodemographic characteristics of the entire cohort and the two clusters.

Characteristic	Overall	Cluster 1	Cluster 2	*p*-value
N	2290	1400	890	
Age, years	11.3 ± 3.6	11.0 ± 3.6	11.9 ± 3.6	<0.001
Sex, n (%)				0.008
Male	1,206 (53%)	706 (50%)	500 (56%)	
Female	1,084 (47%)	694 (50%)	390 (44%)	
Body mass index, kg/m^2^	19.9 ± 4.3	19.4 ± 4.2	20.5 ± 4.5	<0.001
BMI Z-score	0.00 ± 1.00	-0.05 ± 0.97	0.09 ± 1.05	<0.001
Waist circumference, cm	66.8 ± 12.4	65.5 ± 11.8	68.9 ± 12.9	<0.001
WHtR	0.45 ± 0.06	0.45 ± 0.06	0.45 ± 0.06	0.001
WHtR Z-score	0.00 ± 1.00	-0.06 ± 0.97	0.09 ± 1.03	<0.001
General obesity, n (%)	133 (5.8%)	68 (4.9%)	65 (7.3%)	0.019
Abdominal obesity, n (%)	246 (11%)	129 (9.2%)	117 (13%)	0.004
Abdominal obesity by WHtR, n (%)	443 (19.3%)	245 (17.5%)	198 (22.2%)	0.006

Data are presented as the weighted % (standard error) or weighted mean ± standard error.

General obesity, abdominal obesity and abdominal obesity by WHtR were defined as BMI >95^th^ percentile, WC >90^th^ percentile and WHtR ≥0.5, respectively, using the Korean reference data.

WHtR, waist-to-height ratio.

Compared with Cluster 2, Cluster 1 was characterized by a higher frequency of breakfast consumption, higher experience of nutritional education, higher consumption of vegetables, higher consumption of fruits, lower intake of total energy, higher intake of carbohydrate proportion, lower intake of protein proportion, and lower intake of fat proportion. Accordingly, Clusters 1 and 2 were designated as the healthy dietary habit group (HDG) and unhealthy dietary habit group (UDG), respectively. **Table 1** shows the clinical characteristics of Clusters 1 and 2. Participants in Cluster 1 were younger, more likely to be female, less likely to be obese, and had a lower WC.

### 3.2 Association of adiposity with clusters

Based on the linear regression analysis, **Table 2** shows the independent association of BMI, WC, BMI Z-score, and WHtR Z-score with the clusters. Compared with Cluster 1, Cluster 2 had a significantly higher BMI (β-coefficient and 95% confidence interval [CI], 1.06 [0.70–1.42], *p*<0.001), WC (β and 95% CI, 3.41 [2.38–4.44], *p*<0.001), BMI Z-score (β-coefficient and 95% confidence interval [CI], 0.15 [0.06–0.23], *p*<0.001), and WHtR Z-score (β and 95% CI, 1.15 [0.07–0.23], *p*<0.001). **Table 3** shows the cluster-stratified odds ratio (OR) and 95% CI for general obesity and abdominal obesity. Compared with Cluster 1, Cluster 2 had a higher prevalence (OR [95% CI]) of general obesity (1.54 [1.09–2.19], *p*=0.015), abdominal obesity (1.49 [1.14–1.94], *p*=0.003), and abdominal obesity by WHtR (1.35 [1.09-1.66], *p*=0.005). After adjusting for age and sex, Cluster 2 had a significantly higher prevalence (OR [95% CI]) of general obesity (1.49 [1.05–2.13], *p*=0.027), abdominal obesity (1.43 [1.09–1.88], *p*=0.009), and abdominal obesity by WHtR (1.30 [1.05-1.60], *p*=0.018).

**Table 2:** Results of the cluster-stratified linear regression analysis of BMI, WC, BMI Z-score, and WHtR Z-score.

	BMI, Coefficient (95% CI)	*p*-value	WC, Coefficient (95% CI)	*p*-value
Cluster 1	Ref		Ref	
Cluster 2	1.06 (0.70, 1.42)	<0.001	3.41 (2.38, 4.44)	<0.001
	BMI Z-score, Coefficient (95% CI)	*p*-value	WHtR Z-score, Coefficient (95% CI)	*p*-value
Cluster 1	Ref		Ref	
Cluster 2	0.15 (0.06, 0.23)	<0.001	1.15 (0.07, 0.23)	<0.001

BMI, body mass index; WC, waist circumference; WHtR, waist-to-height ratio.

**Table 3:** Results of the cluster-stratified logistic regression analysis for general and abdominal obesity.

	General obesity, OR (95% CI)	*p*-value	Abdominal obesity, OR (95% CI)	*p*-value	Abdominal obesity by WHtR, OR (95% CI)	*p*-value
Unadjusted						
Cluster 1	Ref		Ref		Ref	
Cluster 2	1.54 (1.09-2.19)	0.015	1.49 (1.14-1.94)	0.003	1.35 (1.09-1.66)	0.005
Age-and sex-adjusted						
Cluster 1	Ref		Ref		Ref	
Cluster 2	1.49 (1.05-2.13)	0.027	1.43 (1.09-1.88)	0.009	1.30 (1.05-1.60)	0.018

General obesity, abdominal obesity and abdominal obesity by WHtR were defined as BMI >95^th^ percentile, WC> 90^th^ percentile and WHtR ≥0.5, respectively, using the Korean reference data.

Ref, Reference; WHtR, waist-to-height ratio.

**Figure 3:**
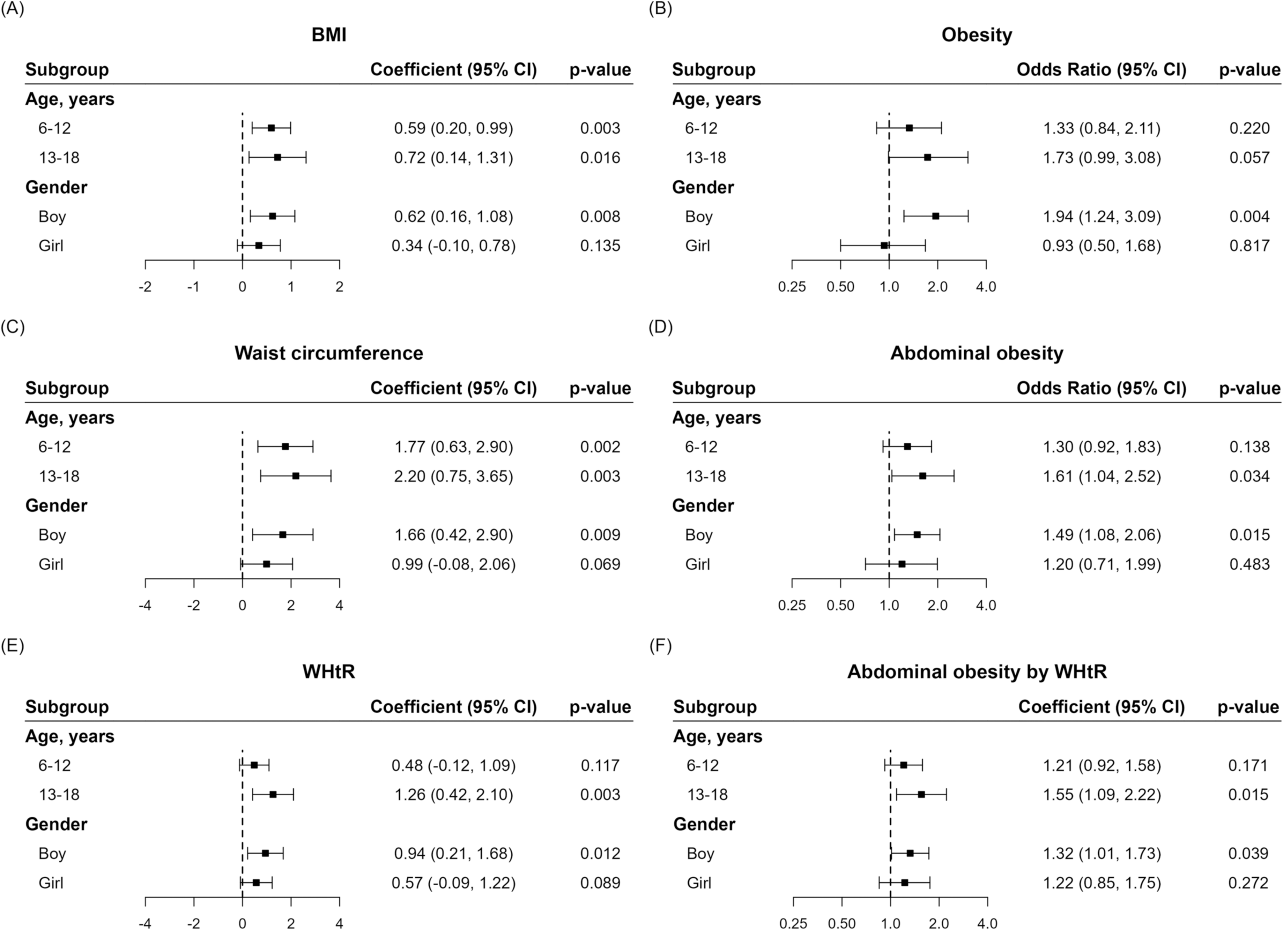
Results of subgroup analysis of anthropometric parameters and obesity indices between Clusters 1 and 2. **(A)** BMI, **(B)** Obesity, **(C)** Waist circumference, **(D)** Abdominal obesity, **(E)** WHtR, **(F)** Abdominal obesity by WHtR).

**Figure 3** presents the results of the age- and sex-stratified subgroup analysis. In both the 6–12 and 13–18 years age groups, Cluster 2 exhibited higher BMI and WC levels than Cluster 1, although the WHtR levels were only significant in the 13-18 years age group. Among boys, compared with Cluster 1, Cluster 2 had higher BMI, WC, and WHtR levels; however, among girls, no significant associations between BMI, WC, WHtR and clusters was observed. Although no significant association was found between general obesity or abdominal obesity and clusters in the 6–12 years age group, in the 13–18 years age group, Cluster 2 exhibited significant trends with a higher prevalence (OR [95% CI]) of general obesity (1.73 [0.99–3.08], *p*=0.057), a significantly higher prevalence of abdominal obesity (1.61 [1.04–2.53], *p*=0.034), and abdominal obesity by WHtR (1.55 [1.09-2.22], *p*=0.015). Among boys, compared with Cluster 1, Cluster 2 had significantly higher prevalence (OR [95% CI]) of general obesity (1.94 [1.24–3.09], *p*=0.004), abdominal obesity (1.49 [1.08–2.06], *p*=0.015), and abdominal obesity by WHtR (1.32 [1.01-1.73], *p*=0.039).

## 4 Discussion

The present study investigated the association between dietary habits and adiposity indices, while particularly focusing on general and abdominal obesity, among Korean children and adolescents. Overall, our findings indicate that individuals exhibiting HDG, such as increased breakfast consumption, greater exposure to nutritional education, and higher fruit and vegetable intake, demonstrated a lower prevalence of childhood obesity.

It is worth noting that 73.6% of children and adolescents in Cluster 1 had breakfast “almost every day,” compared with 59.1% in Cluster 2. A meta-analysis comprising 45 observational studies reported an association between breakfast skipping with overweight/obesity and an increased risk of overweight/obesity (17). Another study demonstrated that frequent breakfast skipping was associated with higher odds of MetS in Korean young adults (18). Mengzi et al. (19) Also found that skipping breakfast was positively associated with both the dietary inflammatory index and obesity, and that the association between eating breakfast and BMI was mediated by the dietary inflammatory index. Our findings align with those of these previous studies. Several possible mechanisms may mediate the association of breakfast consumption with metabolic disturbances. Firstly, owing to increased sleep demand, the overnight fasting periods are longer during childhood and adolescence, leading to the overnight depletion of glycogen stores (20). Consequently, given their higher metabolic rates, breakfast consumption becomes crucial for glucose metabolism in children. Furthermore, skipping breakfast can impair insulin function, resulting in higher postprandial plasma glucose levels (21), which potentially explains why a decreased weekly breakfast frequency is associated with a higher risk of insulin resistance in Korean adults without diabetes or prediabetes (22). Secondly, consistent meal patterns can support better appetite control and satiety, thereby reducing the likelihood of overeating or snacking on less nutritious foods (23, 24). Compared with individuals who regularly consume breakfast, young adults who frequently skip breakfast tend to report higher levels of appetite and hunger, decreased feelings of fullness, and increased ghrelin levels (23, 24). Additionally, breakfast skippers often tend to consume larger amounts of food in one sitting during the remainder of the day (25).

In Cluster 1, 52% of individuals ate vegetables more than twice a day, and 41% ate fruit more than six times a week; compared with Cluster 2, these proportions were significantly higher. It has been well-known that fruits and vegetables reduce the risk of chronic health conditions, including obesity (25–27). One systematic review of cohort studies revealed that higher vegetable intake was associated with the lowest risk of weight gain (28), which is consistent with our findings. Thus, we inferred that fruit and vegetable intake aids weight management because these foods are low in energy, but have high fiber and water content, which induces satiety (29). For children aged 6–11 years, the Dietary Reference Intakes for Koreans (2020) suggested the ideal frequency of fruit and vegetable consumption as once a day (total 300 g/day) and 6–7 cups per day (70 g per cup, total 350 g/day), respectively; for teenagers (12–18 years), vegetables and fruit consumption recommendations were 7–8 cups per day (total 500–550 g/day) and 2–4 times a day (total 200–400 g/day), respectively (30). Although the highest frequency of fruits and vegetables intake were significantly more prevalent in Cluster 1 than in Cluster 2, these were nonetheless actually lower than the abovementioned recommended intake standards. Therefore, we infer that, even if Korean children and adolescents do not meet the intake standards, it is still important to frequently consume fruits and vegetables to prevent obesity, and this can be suggested as a bridging step before aiming to meet the recommended intake standards.

Regarding energy intake, compared with Cluster 2, Cluster 1 consumed less calories, with a higher proportion of carbohydrate and lower proportions of protein and fat. Specifically, compared to the average total energy intake of 1,845 ( ± 694.1) kcal/day in Cluster 1, Cluster 2 had a significantly higher intake of 2,236 ( ± 928.5) kcal/day. In Cluster 1, the proportions of carbohydrates, protein, and fat were 63.9%, 13.8%, and 21.6%, respectively, which are all within the recommended Korean nutrient intake standards (carbohydrates, 55–65%; protein, 7–20%; and fat, 15–30%) for those aged 6–18 years (30, 31). In contrast, Cluster 2 had lower carbohydrate (48.6%) and higher fat (33.1%) intake proportions compared to the national standards. These findings may be associated with the higher consumption of fruits and vegetables in Cluster 1. Foods high in fat, such as meat or fried fast food, are also commonly associated with childhood obesity (31).

Furthermore, we observed that, compared with Cluster 2, Cluster 1 comprised a greater number of children and adolescents who received nutritional education. School-based interventions can effectively reduce the BMI of children (32) and, when implemented in the home, can even improve the BMI of the parents (33). Moreover, this intervention was conducted with only preschool children and favored the prevention of overweight/obesity (34). Nutritional education can be an effective intervention among children as it increases awareness about the importance of food and its impact on overall well-being, which thereby affects overall dietary behaviors. A study found that adolescents who received nutritional education consumed more vegetables and fruits and skipped breakfast less often (35).

In the subgroup analysis, the difference in the prevalence of obesity between the two clusters was markedly evident in a specific age group (13-18 years) and sex (boys). The 13–18 years age group is notable as it is the timepoint at which Korean girls (12.7 years) and boys (13.8 years) reach puberty (36). Obesity occurs during this transitional period at a higher rate (37) because of metabolic changes, including hormonal impact, lifestyle changes, and pubertal stressors (38). Therefore, it seems important to dedicate adequate care to pubertal diet for obesity prevention.

In addition, compared with Korean female adolescents, Korean male adolescents tend to have higher obesity prevalence (39, 40), which aligns with our study results, as well as global statistical trends in high income countries (41). Some studies consider dietary preference as one of the reasons for this difference, thereby indicating that girls, especially in wealthier nations, might favor foods with lower energy content and higher nutrient density, such as fruits and vegetables, whereas boys tend to opt for more calorie-dense foods, such as meat (42, 43). Moreover, compared to boys, girls often express greater weight-related concerns, including the desire to lose weight, feeling of guilt on overeating, and lower self-esteem (44). Parents also typically exhibit more apprehension regarding their daughters’ weight status than that of their sons’, with sons often being encouraged to consume more food (45). These social influences on dietary habits may also explain the sex difference.

Our study has several limitations. Firstly, it is important to consider the impact of the COVID-19 pandemic on the collected data, as our data from the KNHANES VIII (2019-2021) coincided with this period. During the pandemic, South Korea experienced lockdowns and school closures, which contributed to changes in physical activity and dietary habits and a rise in childhood obesity rates (46). Indeed, several studies reported an increase in childhood obesity in Korea during the pandemic, particularly among male students, among whom the prevalence of obesity increased more sharply compared to before the pandemic (47, 48). Additionally, fast food and fruit consumption both decreased (48, 49). These findings partially align with our study results, and suggest that the COVID-19 pandemic likely influenced our data. As such, it is essential to consider the complex effects of the pandemic when interpreting our results. Secondly, the frequency of dietary habits was not assessed with regard to specific intake frequencies, but was rather categorized into sections. Thirdly, the specific foods from which nutrients were obtained could not be determined. Finally, it is important to note that the majority of meals consumed by children are provided by families and educational institutions, rather than being based on their own choices. As such, the evaluation of parental eating habits or the quality of school meals could be beneficial additions to future research endeavors. Despite these limitations, our study has noteworthy strengths. This is the first study to utilize a clustering algorithm to identify dietary behaviors that affect childhood obesity within a large, representative Korean population. By examining nine key dietary variables across distinct clusters, we paved the way for the development of personalized interventional strategies.

In conclusion, distinct clusters that represent different childhood obesity-associated dietary habits were identified. Individuals with healthier dietary behaviors, including increased breakfast consumption, greater exposure to nutritional education, and higher fruit and vegetable intake, exhibited a lower prevalence of childhood obesity. It is also imperative to maintain optimal dietary quality and patterns to effectively prevent childhood obesity. This study underscores the significant role of school and family-based nutritional education and dietary interventions for promoting healthier eating habits among children.

## Data Availability

Publicly available datasets were analyzed in this study. This data can be found here: https://knhanes.cdc.go.kr/knhanes/eng.
